# Family Presence During Resuscitation: Perspectives of Saudi Emergency Medicine Providers

**DOI:** 10.7759/cureus.68218

**Published:** 2024-08-30

**Authors:** Iyad S Kinsarah, Nawaf A AlZahrani, Amro M Gaafar, Anas F Hamam

**Affiliations:** 1 Emergency Medicine, King Fahad Armed Forces Hospital, Jeddah, SAU

**Keywords:** critical, closure, emergency, resuscitation, family

## Abstract

Background

Family presence during resuscitation (FPDR) has been a long-debated topic in medical circles in the last 40 years. Studies usually concentrate on the opinions and responses of the family members of the patients being resuscitated. Only a handful of studies have focussed on the medical practitioner’s opinions and beliefs on the topic. Hence, this study aimed to investigate opinions and beliefs regarding FPDR among emergency medicine practitioners (EMPs) in the Kingdom of Saudi Arabia.

Methodology

This was a self-filled, questionnaire-based study conducted among 450 EMPs across the Kingdom of Saudi Arabia. Proportions and subgroup analyses were conducted with respect to gender, experience, and seniority. EMPs were asked about the number of family members they would allow to attend the resuscitation of a patient under their care in different clinical scenarios.

Results

A total of 392 (87.1%) EMPs participated in this study. Overall, 64% (n = 250) of EMPs reported that they would not allow FPDR in cases that involved trauma, or if the patient being revived was female. This percentage dropped to about 33% (n = 129) when the patient being resuscitated was a child or in out-of-hospital arrest. The majority of EMPs believed that the decision to allow FPDR was the physician’s decision (n = 251, 64.8%) and not the right of the family (n = 133, 34.7%). Moreover, emergency medicine board-certified physicians were more likely to allow FPDR in different cases with 19 (18.5%) in adult arrest cases, 18 (18%) in pediatric cases, and 16 (15.1%) in trauma cases, than non-board-certified practitioners (p = 0.001, 0.007, and 0.031, respectively). Female EMPs were more likely to refuse FPDR than their male counterparts (p < 0.001).

Conclusions

Approximately, 60-70% of EMPs allowed a member of the family to attend the resuscitation of their loved one. However, this percentage dropped in cases where the patient was a female, or the case was a trauma case instead of a medical case. Most Saudi EMPs believed that FPDR hurts the resuscitating team and might hinder the resuscitation efforts.

## Introduction

Many family members and relatives insist on being present when their loved ones are being resuscitated. Family presence during resuscitation (FPDR) is a topic that has been debated back and forth for the last 40 years in the medical literature [1]. On the one hand, there are the relatives of the patient, who are already emotionally distressed, worried, and fearful of what would become of their loved one, as resuscitative efforts being applied by skilled medical practitioners are the only barriers separating life from death. On the other hand, there are medical practitioners, who need to quickly and efficiently resuscitate the patient and save their life and would rather accomplish the task in an environment free of prying, judging layman’s eyes.

The benefits of FPDR are mostly for the relatives and family of the patient. They want closure, the chance to be by their loved ones during this critical time (especially if this is to be the end), and to witness the efforts being made to save the patient (be this by a subconscious act, or a conscious deliberate one) [2]. Although FPDR is the right of the family, studies have shown that most relatives and family members do not want to attend such events or witness such graphic and vivid efforts being applied to their dying patient [3,4]. In a study conducted by Metzger et al., family members who attended their relative’s resuscitation efforts were found to develop major depressive disorder, anxiety, and post-traumatic stress disorder (PTSD) 6.71 times more than those who did not attend [5]. Conversely, in a 2017 Saudi Arabian study, Alshaer et al., reported that 60.9% of relatives said that they would want to be present during the resuscitation of their loved ones and would want their family to be present if ever they were in the unfortunate position to need resuscitation [6].

Opinions of emergency medicine practitioners (EMP) on FPDR have been mixed. Some medical practitioners reported FPDR as a positive experience [7,8], while others reported it as a negative experience [9-11]. However, in a previous study by Goldberger et al., covering 252 hospitals and 41,568 cases of cardiac arrest, FPDR was shown to have no impact on resuscitation outcomes [12]. No study has previously examined EMP opinions concerning this topic in the Middle East region, especially those in Saudi emergency medicine departments. To that end, we aimed to investigate EMP’s viewpoints on this controversial topic. Hence, this study aimed to investigate opinions and beliefs regarding FPDR among EMPs in the Kingdom of Saudi Arabia (KSA).

## Materials and methods

Study design and setting

This cross-sectional survey was conducted from December 15, 2023, to June 1, 2024, using a self-administered online questionnaire, which was distributed through email to 450 EMPs in Saudi Arabian emergency departments.

Study population

This study included residents in the emergency medicine residency training program, emergency medicine board-certified (EMBCP) physicians, and general practitioners (GPs) working in the emergency department. Consent for participation was obtained through the questionnaire.

Eligibility criteria

For inclusion in the study, the participant needed to be a physician currently working in an emergency medicine department in KSA. The participant needed to be a resident in the emergency medicine residency training program, EMBCP, or GP working in an emergency medicine department in KSA. All non-physician staff, such as nurses and paramedic technicians, were excluded. Further, all physicians not meeting the above-mentioned criteria (i.e., not currently working, working in a primary care center, etc.) were also excluded.

Data collection tool

A self-administered online questionnaire was used to collect data from the included participants [12]. The survey was divided into two main parts. The first collected the demographic data of the participants, including age, gender, years of practice, and emergency medicine certification. The second and main topic of the study was to ask EMPs about their opinions of the number of family members/relatives they would usually allow to attend the resuscitation on a patient under their care if the patient was an adult versus a child, and if the case was a medical condition versus a trauma case.

Sample size calculation

For a type I error of 0.05 (alpha), an expected proportion of 0.5 (p), an absolute error or precision (d) of 0.05, and a population of about 1,500 emergency medicine physicians, the required sample size, as calculated by an online calculator (https://www.calculator.net/sample-size-calculator.html), was 306 participants.

Sampling technique

The random sampling technique was used for the study. The names of 25 major emergency departments in KSA were placed in a randomizing site, and the first 15 names had the survey emailed to their EMPs to collect data from the participants.

Data analysis

After data collection, it was entered into a single database and analyzed using SPSS version 26 (IBM Corp., Armonk, NY, USA). Proportions were noted, and the Mann-Whitney Z test and Kruskal Wallis H test were used to measure sociodemographic data. Due to the non-normal distribution of data, the non-parametric Pearson correlation test was applied for all subgroup analyses to ascertain the statistical significance of the findings, concerning gender, years of practice, and certification.

Ethical considerations

This study was approved by the Research Ethics Committee of Armed Forces Hospitals, Jeddah (approval code REC635 issued on November 26, 2023). All data were kept confidential and used only for research purposes.

## Results

The survey was sent to 450 EMPs throughout KSA via email. In total, 414 (92%) responded, of whom only 392 (87.1%) returned completed questionnaires and were included in the data analysis. The sample comprised 69.6% (n = 273) males. The participating physicians included 278 (70.1%) residents, and 103 (26.3%) EMBCPs. Participating physicians who were practicing for more than five years in the field made up 25.6% (n = 100) of the study sample (Table 1).

**Table 1:** Demographics of the study participants. The data are presented as frequency (n) and percentage (%). EM = emergency medicine; EMBCP = emergency medicine board-certified physician; PGY = program year

Data	n (%)
Gender	
Male	273 (69.6)
Female	119 (30.4)
Years of practice	
Less than 3 years	165 (42.1)
3–5 years	127 (32.3)
6–10 years	50 (12.8)
More than 10 years	50 (12.8)
Participant medical discipline	
General practitioner	11 (2.6)
EM resident	278 (70.1)
EMBCP	103 (26.3)
Participant level of training	
None	11 (2.8)
PGY 1	120 (30.6)
PGY 2	63 (16.1)
PGY 3	47 (12.0)
PGY 4	48 (12.2)

**Figure 1:**
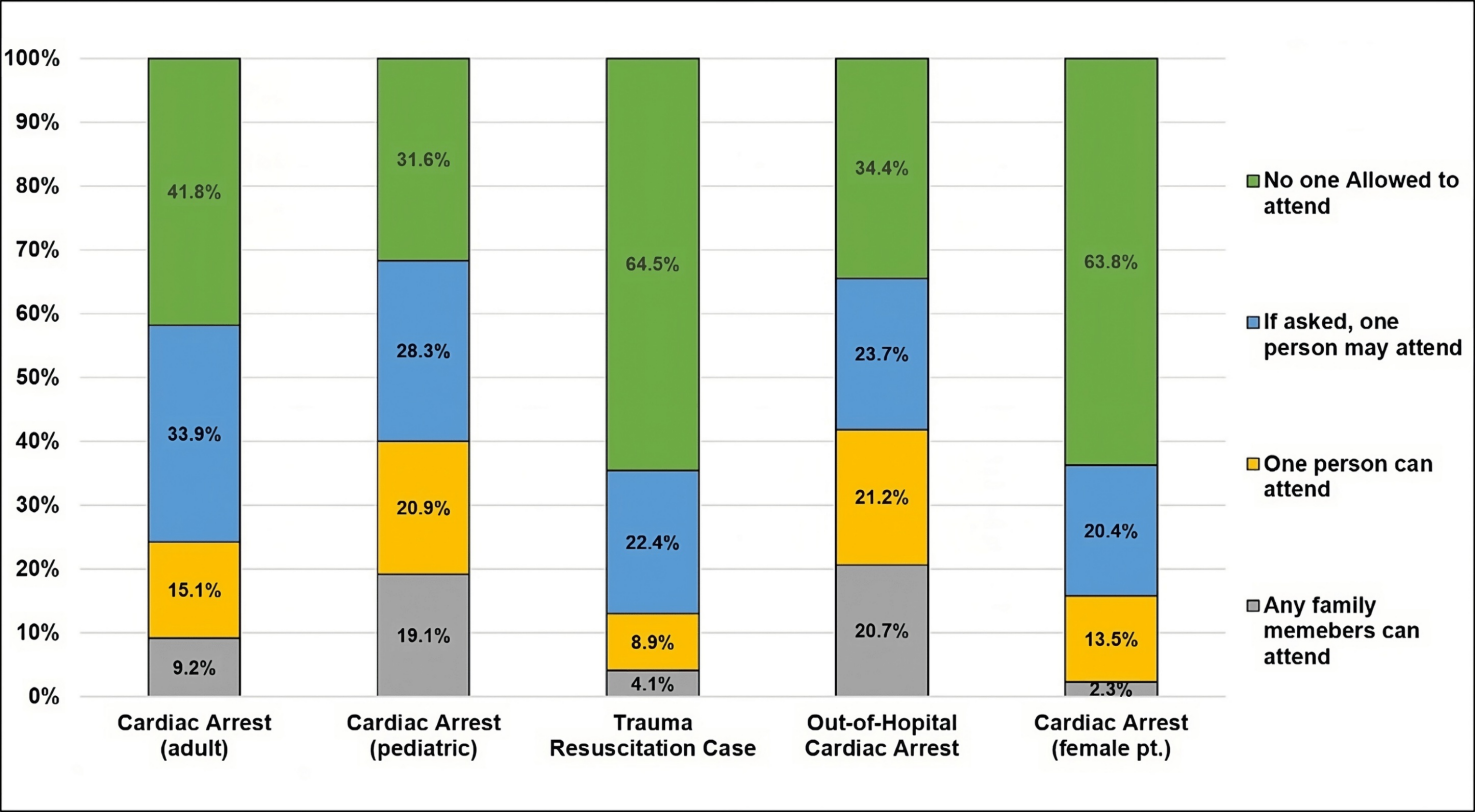
Allowing FPDR in different resuscitation settings. FPDR = family presence during resuscitation

When asked about FPDR in an in-hospital cardiac arrest resuscitation, 164 (41.8%) said they would not allow anyone to attend the resuscitation, and 133 (33.9%) said they would only allow a single person if they were asked to do so. In contrast, in pediatric cases, and out-of-hospital arrest cases, an almost 10% shift in responses was seen, with only 31-34% of EMPs stating that they would not allow any attendees. Of note, regarding the response in cases of trauma, or cases involving female patients (as the patient might be excessively exposed during the resuscitation protocol), nearly 251 (64%) EMPs in both cases would not allow any attendees (Figure 1).

**Figure 2:**
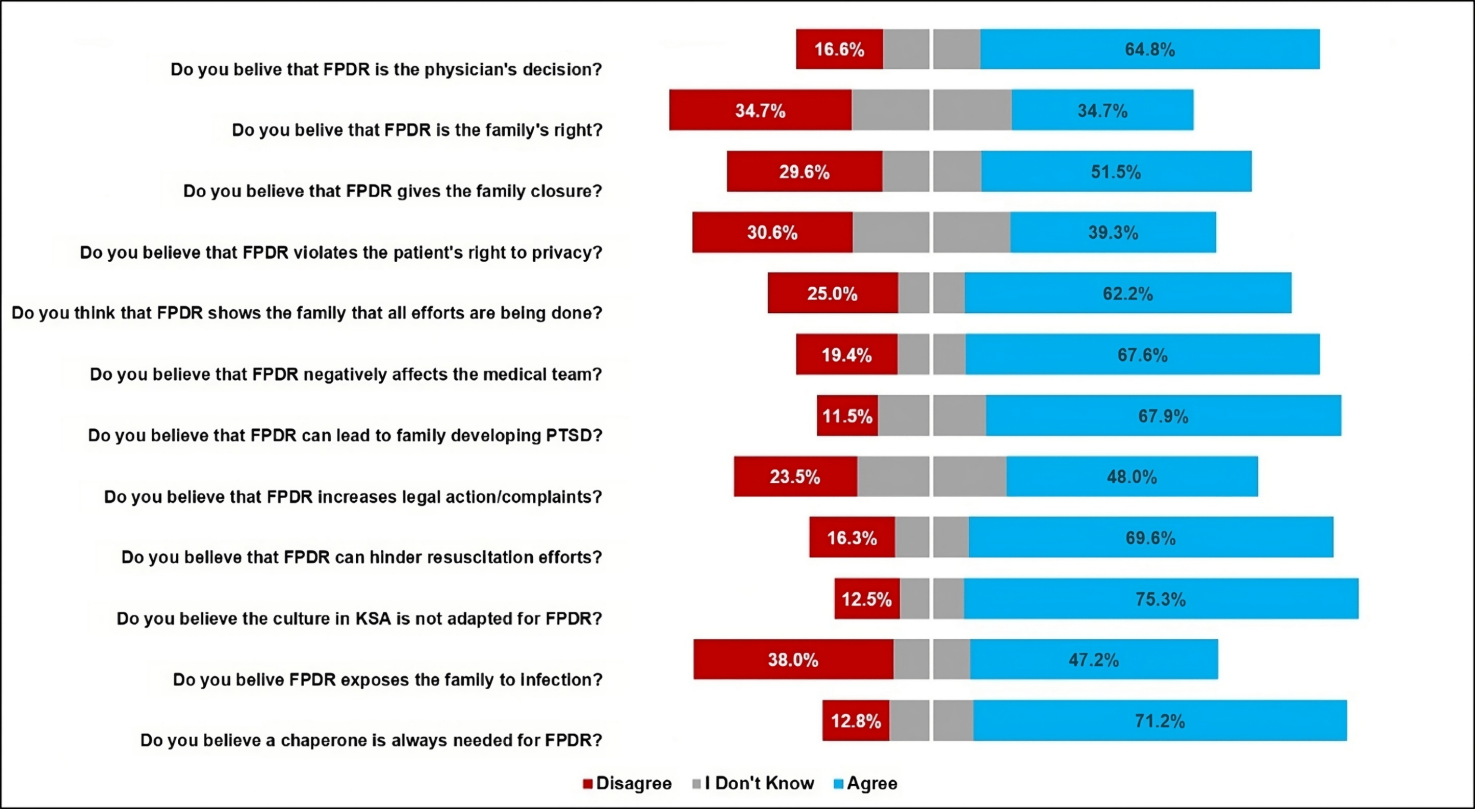
Opinions of emergency medicine practitioners on FPDR issues. FPDR = family presence during resuscitation; PTSD = post-traumatic stress disorder

EMPs who participated in this study were asked several questions about their opinions regarding FPDR. The majority of EMPs believed that the decision to allow FPDR was the physician’s decision (n = 279, 71.2%) and not the right of the family (n = 149, 38%) (Figure 2).

When EMPs were asked about their beliefs regarding FPDR, 188 (48%) EMPs agreed that FPDR shows the family that all efforts are being made, 266 (67.9%) agreed that FPDR negatively affects the medical team, 265 (67.6%) agreed that FPDR can lead to the family developing PTSD, and 154 (39.3%) agreed on FPDR likely hindering resuscitation efforts (Table 2).

**Table 2:** The beliefs of emergency medicine practitioners about FPDR. The data are presented as frequency (n) and percentage (%) and mean and standard deviation (SD). FPDR = family presence during resuscitation; PTSD = post-traumatic stress disorder

Question	Agree, n (%)	Don’t know, n (%)	Disagree, n (%)	Mean	SD	Final EMP response
Do you believe that FPDR is the physician’s decision?	279 (71.2)	63 (16.1)	50 (12.8)	3.666	0.057	Agree
Do you believe that FPDR is the family’s right?	185 (47.2)	58 (14.8)	149 (38)	2.977	0.055	Disagree
Do you believe that FPDR gives the family closure?	295 (75.3)	48 (12.2)	49 (12.5)	3.260	0.058	Disagree
Do you believe that FPDR violates the patient’s right to privacy?	273 (69.6)	55 (14)	64 (16.3)	3.110	0.053	Disagree
Do you think that FPDR shows the family that all efforts are being made?	188 (48)	112 (28.6)	92 (23.5)	3.508	0.060	Agree
Do you believe that FPDR negatively affects the medical team?	266 (67.9)	81 (20.7)	45 (11.5)	3.732	0.059	Agree
Do you believe that FPDR can lead to the family developing PTSD?	265 (67.6)	51 (13)	76 (19.4)	3.788	0.051	Agree
Do you believe that FPDR increases legal action/complaints?	244 (62.2)	50 (12.8)	98 (25)	3.311	0.053	Disagree
Do you believe that FPDR can hinder resuscitation efforts?	154 (39.3)	118 (30.1)	120 (30.6)	3.727	0.053	Agree
Do you believe the culture in Saudi Arabia is not adapted for FPDR?	202 (51.5)	74 (18.9)	116 (29.6)	3.875	0.052	Agree
Do you believe FPDR exposes the family to infection?	136 (34.7)	120 (30.6)	136 (34.7)	3.130	0.059	Disagree
Do you believe a chaperone is always needed for FPDR?	254 (64.8)	73 (18.6)	65 (16.6)	3.788	0.054	Agree

When asked about FPDR in a child’s resuscitation more male EMPs were willing to allow one or more family members to attend the resuscitation than female EMPs (122 (44.7%) vs. 35 (29.4%), respectively; p = 0.039). When asked about FPDR where the patient was a female, 90 (75.6%) female EMPs said that they would not allow any family member to attend, while 160 (58.6%) male EMPs refused family attendance in such a case (p < 0.001). Additionally, 13 (11.2%) more female EMPs agreed that FPDR brings closure to the family (p = 0.008) than their male counterparts, while 34 (12.4%) more male EMPs said that FPDR also served to demonstrate to the family that all resuscitation efforts are being made to save the patient (p = 0.045). Finally, nine (7.5%) more female EMPs believed that FPDR would result in family members developing PTSD (p = 0.004).

Senior residents were 13% more likely to allow FPDR than junior residents, while more junior residents would not allow FPDR in adult arrest cases (42 (22.8%)) and female resuscitation cases (49 (26.7%)) (p < 0.001). Moreover, in pediatric and traumatic resuscitation cases, juniors were (31 (16.9%) and 34 (18.2%), respectively) more likely to refuse FPDR than seniors (p = 0.002 and 0.016, respectively). Senior residents tended to believe that FPDR is the family’s right 18.7% more than their junior counterparts (p = 0.012). Juniors were 15.5% more likely to believe that FPDR negatively affects the performance of the resuscitation team, and 21 (11.8%) more juniors believed that FPDR hinders resuscitative efforts (p = 0.029 and 0.014, respectively).

Furthermore, EMBCPs were more likely to allow FPDR in different cases: adult arrest cases, 19 (18.5%); pediatric cases, 18 (18%); and trauma cases, 16 (15.1%) than non-board-certified practitioners (p = 0.001, 0.007, and 0.031, respectively). EMBCPs’ responses showed that they believed that FPDR was the family’s right. Moreover, 17 (15.7%) non-certified EMPs and 20 (19.1%) EMBCPs believed that FPDR did not violate the patient’s privacy (p = 0.026 and 0.005, respectively). EMBCPs (n = 17, 15.7%) were less likely to believe that FPDR hurts the resuscitation team’s performance, and 12 (12.1%) were less likely to believe that FPDR hinders resuscitation efforts (p = 0.001 and 0.010, respectively).

## Discussion

The data from this study is interesting as it shines a light on what emergency practitioners thought about the much-debated topic of FPDR. Most EMPs tended to restrict access to the family attending resuscitation efforts in cases of trauma and when the patient was a female. Understandably, seeing a patient being resuscitated in a trauma case where there might be mangled limbs, fractures, and bleeding all over the place has a more shocking psychological impact on a bystander only watching cardiopulmonary resuscitation being performed on a patient with an intact body. Of note, EMPs were more likely to allow more people to attend trauma resuscitation than resuscitation in cases where the patient was female. In Arabic and Islamic countries, such findings do make sense as this is part of the religion and culture of the society.

The data concerning the beliefs of EMPs regarding the different aspects of FPDR also showed some interesting results. While FPDR is the right of the family, most EMPs responded that it was not. The decision to allow FPDR, according to the study data, belongs to the physician in charge, and not the family. This belief does not seem to be built on the notion that FPDR is a violation of the patient’s right to privacy, as most EMPs responded that FPDR does not violate that right. Further, EMPs tended to agree that FPDR hurts the resuscitation team and hinders the resuscitation effort. This is in keeping with previously published evidence [9-11]. However, the belief that FPDR is the decision of the physician does not seem to stem from the fear of an increase in medicolegal complaints, nor that the resuscitating team would be working under “the eyes” of the family. Although most EMPs believed that allowing FPDR would show the family that all efforts are being made, it was also believed that this might cause them to develop PTSD, although this has not been conclusively proven [13,14].

Probably the origins of all of this were the highest percentages found in current data, 295 (75.3 %) EMPs agreed that the Saudi Arabian culture and society are not adapted or oriented to understand what is going on during resuscitation. In the last 30 years, KSA has made giant strides in the fields of education, technical progress, and medicine, bringing the country from tribal medicine into the modern age in an astoundingly short period. Understandably, not many in the general society made the same progress. The general population in KSA is usually less educated on such matters than the populations in other developed countries. It was found that many of that older generation, who lived through hard times when the country was still developing its infrastructure, during the oil boom era, immediately after the end of the Second World War, and who lived most of their lives being treated with tribal medicine, are still alive today. Hence, it 279 (71.2%) of EMPs suggested the need for a chaperone to explain to the attending family members the ongoings of a resuscitation effort.

On subgroup analysis, current data suggested that the more experienced an EMP was, being more senior in their residency training program, or being board-certified in emergency medicine (meaning that they have finished their residency training), the more likely they were to allow FPDR. EMBCPs were less likely to believe that FPDR hurts the resuscitation team or the resuscitation effort itself, which is in keeping with the evidence shown by Goldberger et al. [12]. The finding that male EMPs tended to allow more family members to attend resuscitation than their female counterparts probably stems from the patriarchal nature of Saudi society. Until recently, women in Saudi society were not allowed the same opportunities as men, and, as such, female EMPs might feel more under scrutiny from a patriarchal populous than male EMPs, which is in keeping with previously observed patterns in the FPDR issue [15].

This study had some limitations. The survey was distributed over email, with an 87% response rate. EMPs who did not get the email due to it being shifted to the junk box or those with minimal internet access could not contribute to the study. The study questionnaire was not fully validated. The questions in the survey were collected from different previously used questionnaires. Face validity of the final questionnaire was established by five emergency medicine consultants, but total validation of the survey was not done. Due to differences in cultural issues, the generalization of the study findings might be limited. The study mainly concentrated on obtaining the opinions of EMPs, which cannot be generalized similarly to other resuscitations expected in the in-hospital setting such as anesthesia or intensive care professionals, nor can it be extended to the pre-hospital and paramedical settings.

## Conclusions

Approximately, 60-70% of EMPs would allow a member of the family to attend the resuscitation of their loved one. However, this percentage dropped in cases where the patient was a female, or the case was a trauma case instead of a non-trauma case. Most Saudi EMPs believed that FPDR hurts the resuscitating team and might hinder the resuscitation efforts.
